# Supplementary material for: The use of the phrase “data not shown” in dental research
Source: PLoS One. 2022 Aug 9;17(8):e0272695. doi: 10.1371/journal.pone.0272695 (PMC9362922; doi:10.1371/journal.pone.0272695)
Supplement: S1 Text — (DOCX) [file pone.0272695.s001.docx]

# S1 Text. Deviations from the protocol

After we gathered and analysed data and wrote the manuscript according to protocol, we felt that the manuscript needed some more depth results to complement the manuscript. First, we decided to make an analysis about the longer-term trend in the use of phrases “data/results not shown” in medical and dental articles available from the PubMed Central open-access database. We did that by using these two search terms and then copying the number of returned results for each year into our spreadsheet:

- For all PubMed-indexed open-access articles within a specific year; e.g., 2020:
  - ((IN_EPMC:y) OR (OPEN_ACCESS:y)) AND (SRC:"MED") AND (LANG:"eng" OR LANG:"en" OR LANG:"us") AND (FIRST_PDATE:[2020-01-01 TO 2020-12-31])
- For PubMed-indexed open-access articles with “data/results not shown” within a specific year; e.g., 2020:
  - (“results not shown" OR "data not shown") AND (SRC:"MED") AND (LANG:"eng" OR LANG:"en" OR LANG:"us") AND (FIRST_PDATE:[2020-01-01 TO 2020-12-31])

In addition, we noted that within a few weeks, 18 new articles with the phrases were published in dental journals, and so we removed all publication time restrictions from our search on 29 September when we ran the updated search.

Second, we extracted publisher information for each journal in order to study publisher-related differences in publishing the articles with phrases. The reason why the publisher would matter stems from the fact that some publishers, e.g. The Public Library of Science (PLOS), have forbidden the use of the phrase (<https://journals.plos.org/plosone/s/data-availability>).
