# Supplementary material for: The use of the phrase “data not shown” in dental research
Source: PLoS One. 2022 Aug 9;17(8):e0272695. doi: 10.1371/journal.pone.0272695 (PMC9362922; doi:10.1371/journal.pone.0272695)
Supplement: S2 Text — (DOCX) [file pone.0272695.s002.docx]

# S4 Text. Sensitivity analysis for publisher-related differences

We found that publisher information for journals varied considerably depending on the source. Thus, we searched the name of the publisher of each journal from Publons and for sensitivity analysis also from SCImago and National Library of Medicine, NLM, Catalog. For all those three we ran similar Fisher exact tests for publisher-related differences in publishing studies with the phrases “data/results not shown”.

The results were similar regardless of the source of information. The sensitivity analysis for publisher-related differences resulted in p-values of <0.001 for SCImago and 0.003 for NLM catalogue retrieved publisher information, while for the Publons the test resulted in a p-value of 0.002 (tables shown in supplementary material at osf.io/5zryu/).
