# Supplementary material for: The use of the phrase “data not shown” in dental research
Source: PLoS One. 2022 Aug 9;17(8):e0272695. doi: 10.1371/journal.pone.0272695 (PMC9362922; doi:10.1371/journal.pone.0272695)
Supplement: Attachment — Submitted filename: marked-upPONE-D-21-34697_reviewer.pdf [file pone.0272695.s003.pdf]

PLOS ONE

Movement towards open science: Have dental researchers abandoned the outdated caveat “data not shown”?

--Manuscript Draft--

**Manuscript Number:**	PONE-D-21-34697
**Article Type:**	Research Article
**Full Title:**	Movement towards open science: Have dental researchers abandoned the outdated caveat “data not shown”?
**Short Title:**	Short title: “data not shown” in dental literature
**Corresponding Author:**	Ahmad Sofi-Mahmudi, DDS<br>Kurdistan University of Medical Sciences<br>Saqiz, Kurdistan IRAN, ISLAMIC REPUBLIC OF
**Keywords:**	Dentistry; Data; Open Science
**Abstract:**	Objective<br><br>To investigate how frequently papers published in dental journals use the phrases “data/results not shown” and to what kind of results the authors referred to with these phrases in 2021.<br><br>Methods<br><br>We searched the Europe PubMed Central (PMC) database for open access articles available from studies published in PubMed-indexed dental journals until 29 September 2021. We searched for “data/results not shown” phrases from the full texts and then calculated the proportion of articles with the phrases in all the available articles. From studies published between 1 January 2021 and 29 September 2021, we evaluated whether the phrases referred to confirmatory results, negative results, peripheral results, sensitivity analysis results, future results or other/unclear results. Journal-related differences in publishing studies with the phrases in 2021 were tested with the Fisher’s exact test along with Monte Carlo simulations using the R v4.1.1 software.<br><br>Results<br><br>The percentage of studies with the relevant phrases from the total number of studies in the database decreased from 13% to 3% between 2010 and 2020. In 2021, from 1 951 studies published in 71 different journals, 57 (2.9%) used the phrases. Potential journal-related differences in publishing studies with the phrases were detected in 2021 (p=0.03). Most commonly, the phrases referred to negative (n=17, 30%), peripheral (n=14, 24%) or confirmatory (n=9, 16%) results. The significance of unpublished results to which the phrases referred considerably varied across studies.<br><br>Conclusion<br><br>Over the last decade, there has been a marked decrease in the use of the phrases “data/results not shown” in dental journals. However, the phrases were still notably in use in dental studies in 2021, despite the good availability of accessible free online supplements and repositories.
**Order of Authors:**	Eero Raittio
	Ahmad Sofi-Mahmudi, DDS
	Erfan Shamsoddin
**Additional Information:**	
**Question**	**Response**
**Financial Disclosure**	The author(s) received no specific funding for this work.

Powered by Editorial Manager® and ProduXion Manager® from Aries Systems Corporation

Enter a financial disclosure statement that describes the sources of funding for the work included in this submission. Review the submission guidelines for detailed requirements. View published research articles from *PLOS ONE* for specific examples.<br><br>This statement is required for submission and **will appear in the published article** if the submission is accepted. Please make sure it is accurate.<br><br>**Unfunded studies**<br>Enter: *The author(s) received no specific funding for this work.*<br><br>**Funded studies**<br>Enter a statement with the following details:<br>• Initials of the authors who received each award<br>• Grant numbers awarded to each author<br>• The full name of each funder<br>• URL of each funder website<br>• Did the sponsors or funders play any role in the study design, data collection and analysis, decision to publish, or preparation of the manuscript?<br>• **NO** - Include this sentence at the end of your statement: *The funders had no role in study design, data collection and analysis, decision to publish, or preparation of the manuscript.*<br>• **YES** - Specify the role(s) played.<br><br>* typeset	
**Competing Interests**<br><br>Use the instructions below to enter a competing interest statement for this submission. On behalf of all authors, disclose any competing interests that could be perceived to bias this work—acknowledging all financial support and any other relevant financial or non-financial competing interests.<br><br>This statement is required for submission and **will appear in the published article** if	The authors have declared that no competing interests exist.

the submission is accepted. Please make sure it is accurate and that any funding sources listed in your Funding Information later in the submission form are also declared in your Financial Disclosure statement.<br><br>View published research articles from *PLOS ONE* for specific examples.<br><br>**NO authors have competing interests**<br><br>Enter: *The authors have declared that no competing interests exist.*<br><br>**Authors with competing interests**<br><br>Enter competing interest details beginning with this statement:<br><br>*I have read the journal's policy and the authors of this manuscript have the following competing interests: [insert competing interests here]*<br><br>* typeset	
**Ethics Statement**<br><br>Enter an ethics statement for this submission. This statement is required if the study involved:<br><br>• Human participants<br>• Human specimens or tissue<br>• Vertebrate animals or cephalopods<br>• Vertebrate embryos or tissues<br>• Field research<br><br>Write "N/A" if the submission does not require an ethics statement.<br><br>General guidance is provided below. Consult the submission guidelines for detailed instructions. **Make sure that all information entered here is included in the Methods section of the manuscript.**	N/A

**Format for specific study types**<br><br>**Human Subject Research (involving human participants and/or tissue)**<br>• Give the name of the institutional review board or ethics committee that approved the study<br>• Include the approval number and/or a statement indicating approval of this research<br>• Indicate the form of consent obtained (written/oral) or the reason that consent was not obtained (e.g. the data were analyzed anonymously)<br><br>**Animal Research (involving vertebrate animals, embryos or tissues)**<br>• Provide the name of the Institutional Animal Care and Use Committee (IACUC) or other relevant ethics board that reviewed the study protocol, and indicate whether they approved this research or granted a formal waiver of ethical approval<br>• Include an approval number if one was obtained<br>• If the study involved *non-human primates*, add *additional details* about animal welfare and steps taken to ameliorate suffering<br>• If anesthesia, euthanasia, or any kind of animal sacrifice is part of the study, include briefly which substances and/or methods were applied<br><br>**Field Research**<br><br>Include the following details if this study involves the collection of plant, animal, or other materials from a natural setting:<br>• Field permit number<br>• Name of the institution or relevant body that granted permission	
**Data Availability**<br><br>Authors are required to make all data underlying the findings described fully available, without restriction, and from the time of publication. PLOS allows rare exceptions to address legal and ethical concerns. See the PLOS Data Policy and FAQ for detailed information.	Yes - all data are fully available without restriction

A Data Availability Statement describing where the data can be found is required at submission. Your answers to this question constitute the Data Availability Statement and **will be published in the article**, if accepted.<br><br>**Important:** Stating 'data available on request from the author' is not sufficient. If your data are only available upon request, select 'No' for the first question and explain your exceptional situation in the text box.<br><br>Do the authors confirm that all data underlying the findings described in their manuscript are fully available without restriction?	
**Describe where the data may be found in full sentences. If you are copying our sample text, replace any instances of XXX with the appropriate details.**<br><br>• If the data are **held or will be held in a public repository**, include URLs, accession numbers or DOIs. If this information will only be available after acceptance, indicate this by ticking the box below. For example: *All XXX files are available from the XXX database (accession number(s) XXX, XXX.*).<br>• If the data are all contained **within the manuscript and/or Supporting Information files**, enter the following: *All relevant data are within the manuscript and its Supporting Information files.*<br>• If neither of these applies but you are able to provide **details of access elsewhere**, with or without limitations, please do so. For example:<br><br>*Data cannot be shared publicly because of [XXX]. Data are available from the XXX Institutional Data Access / Ethics Committee (contact via XXX) for researchers who meet the criteria for access to confidential data.*<br><br>*The data underlying the results presented in the study are available from (include the name of the third party*	The data underlying the results presented in the study are available from https://osf.io/5zryu.

and contact information or URL).<br>• This text is appropriate if the data are owned by a third party and authors do not have permission to share the data.<br><br>* typeset	
Additional data availability information:	

Manuscript

Click here to access/download;Manuscript;Manuscript - Data Availability - PLOS ONE.docx

Movement towards open science: Have dental researchers abandoned the outdated caveat "data not shown"?

Short title: "data not shown" in dental literature

Eero Raittio[1], Ahmad Sofi-Mahmudi[2*], Erfan Shamsoddin[2]

[1] Institute of Dentistry, University of Eastern Finland, Kuopio, Finland.

[2] Cochrane Iran Associate Centre, National Institute for Medical Research Development (NIMAD), Tehran, Iran.

Corresponding author:

Ahmad Sofi-Mahmudi; Address: 21, National Institute for Medical Research Development (NIMAD), West Fatemi St., Tehran, Tehran, Iran; Postal code: 1419693111; Telephone: +98 21 6693 8037; Email: sofimahmudi@research.ac.ir, a.sofimahmudi@gmai.com.

Abstract

Objective: To investigate how frequently papers published in dental journals use the phrases “data/results not shown” and to what kind of results the authors referred to with these phrases in 2021.

Methods: We searched the Europe PubMed Central (PMC) database for open access articles available from studies published in PubMed-indexed dental journals until 29 September 2021. We searched for “data/results not shown” phrases from the full texts and then calculated the proportion of articles with the phrases in all the available articles. From studies published between 1 January 2021 and 29 September 2021, we evaluated whether the phrases referred to confirmatory results, negative results, peripheral results, sensitivity analysis results, future results or other/unclear results. Journal-related differences in publishing studies with the phrases in 2021 were tested with the Fisher’s exact test along with Monte Carlo simulations using the R v4.1.1 software.

Results: The percentage of studies with the relevant phrases from the total number of studies in the database decreased from 13% to 3% between 2010 and 2020. In 2021, from 1 951 studies published in 71 different journals, 57 (2.9%) used the phrases. Potential journal-related differences in publishing studies with the phrases were detected in 2021 (p=0.03). Most commonly, the phrases referred to negative (n=17, 30%), peripheral (n=14, 24%) or confirmatory (n=9, 16%) results. The significance of unpublished results to which the phrases referred considerably varied across studies.

Conclusion: Over the last decade, there has been a marked decrease in the use of the phrases “data/results not shown” in dental journals. However, the phrases were still notably in use

in dental studies in 2021, despite the good availability of accessible free online supplements and repositories.

Keywords: Dentistry; Data; Open Science

Introduction

The foundation of science and research is sustainable, valid and reliable when the results are available to be tested, replicated and reproduced [1–4] Open access articles, data and code sharing, funding and conflicts of interest disclosures and detailed descriptions of materials, methods and results are great facilitators to open science [4–6]. However, studies suggest that too frequently published results may be non-reproducible [5,7–9]. Fortunately, open science practices have also been adopted in biomedical research over the last decades [5,10]. For instance, some journals have adopted compulsory data and code availability statements and abandoned strict word, table and figure limits. Additionally, online repositories and scientific publishers' online supplements to articles have facilitated easy and free data as well as code and document sharing. Open Science Framework (OSF, www.osf.io), figshare (www.figshare.com) and GitHub (www.github.com) are some examples of online platforms where one can manage project information and data/code sharing and archive for free.

Frequently, phrases such as "data not shown" or "results not shown" are used to refer to unpublished results. These results may be related to confirmatory analyses (similar results published elsewhere), negative results, peripheral results (not directly related to the topic), sensitivity analyses or future results (e.g., results related to the manuscript in preparation) [11]. In agreement with the endeavour towards transparent and reproducible science, such phrases can be seen as "an outdated caveat that obscures the transparency of a scientific report and weakens the peer review process" [12]. Some journals and publishers have even forbidden their use [12]. However, it remains unknown how frequently published studies

include such phrases to refer to unpublished results in the current era of open access online journals and free online repositories and supplements to articles.

Therefore, we aimed to investigate how frequently papers published in dental journals used the phrases “data/results not shown”. Accordingly, from relevant studies in 2021, we examined what kind of results the authors referred to with these phrases. Further, we also examined the data sharing statements, data, supplement and code availability for the studies that used these phrases.

Materials and Methods

Study selection

We registered the protocol for this study on OSF on 26 September 2021 (osf.io/5zryu). All codes and data are also available on osf.io/5zryu. Deviations from the protocol are available in S1 Text. We conducted searches in the Europe PubMed Central (PMC) database, which contains over seven million full-text articles at the moment. First, we searched all PMC open access articles (PMCOA) published in the PubMed-indexed dental journals in the database until 29 September 2021 (S2 Text). Then, we searched for the phrases “data not shown” and “results not shown” from the PMCOA articles published in the same journals until the same date (S3 Text).

From those searches, we selected studies published between 1 January 2021 and 29 September 2021. From studies for which the search indicated that they included the phrases, we manually confirmed whether the phrase referred to unreported/unpublished results. If they did so, we included them for further analysis.

Data extraction and synthesis

From studies published in 2021 with the "data/results not shown" phrases, we documented whether the study shared data or code or online supplementary materials/appendices within the journal website or via other platforms. Then, we categorised the studies based on whether the "data/results not shown" phrases referred to confirmatory results, negative results, peripheral results, sensitivity analysis results, future results or other/unclear categories (Table 1) [11]. We also searched for information about publishing free online supplementary materials from the websites of all journals which had published at least one paper in the subsample (yes unlimited, yes limited, unclear, no). We searched the name of the publisher of each journal from Publons (and for sensitivity analysis also from SCImago and National Library of Medicine, NLM, Catalog). All data extractions from full texts were performed first by one of the authors, and all extractions were confirmed/checked by another author; discrepancies were solved through discussion.

Table 1. Definitions of phrase types with examples, adapted from [11].

Type	Definition	Example from the sample
Confirmatory results	Demonstrating the reproducibility of previous findings or demonstrating	"The results for the negative control group consistently showed no bacterial growth (data not shown)" [30].

	validity methods (e.g., negative controls).	
Negative results	Results that did not show statistically significant effect/association on some threshold, e.g., p > 0.05.	“No adhesive changed IL-10 and TNFα expression at any time point (data not shown)” [31].
Sensitivity analysis results	Sensitivity analyses assess the robustness of results, for instance, the impact of including or excluding some variables from the analysis.	“In addition, sensitivity analyses conducted, limiting the data to those with known values of isoprostanes and plasma carbonyls, yielded similar results (data not shown)” [32].
Peripheral results	Referring to results not directly relevant to the main topic of article, often mentioned in the discussion section.	“Results of different studies revealed moderate correlations between the MDAS [Modified Dental Anxiety Scale] and dentists’ observations (0.4 to 0.66) [9, 34, 37]. In our study, the strength of the correlation between the dentist’s observations and MDAS scoring was also moderate (results not shown)” [33].
Future results	Results which are going to be represented in other subsequent papers, often mentioned in the discussion.	Not in the sample
Other/unclear	For instance, related to statistical procedure selection (e.g., normality tests), studies with multiple phrases and varying purposes, and phrases with vague purposes.	“Since the material includes methacrylates, its potential for adverse side effects was additionally evaluated in the present study (data not shown)” [34].

For each publication year, we calculated the percentage of studies with the “data/results not shown” phrases from the total number of PMCOA articles published in the same dental

journals during the year. We also searched the phrases from all PubMed-indexed PMCOA (S1 Text) and wrote down the returned number of hits for each year to make a comparison with PMCOA articles from dental journals. We reported our findings with simple descriptive tables and figures, as well as provided some examples of how the phrases were used. Journal- and publisher-related differences in the number of studies with the phrase, from the total number of PMCOA articles from each journal or publisher in 2021, were tested using the Fisher's exact test with Monte Carlo simulations, with 100 000 replications. We used the R v4.1.1 software (2021-08-10, R Foundation for Statistical Computing, Vienna, Austria. http://www.R-project.org) for statistical analysis. According to the latest statement by the American Statistical Association, no statistically significant level was considered in our analyses [13].

Results

The search from the Europe PMC database identified 18 760 unique PMCOA articles from 113 different dental journals until 29 September 2021. From these, the search for "data/results not shown" phrases produced 1 389 unique records from 67 various dental journals. The percentage of studies with the phrases from all the PMCOA articles was seen to have decreased from 13% to approximately 3% in dental journals from 2010 to 2020 (Figure 1A). The same downtrend was seen amongst all PubMed-indexed PMCOA articles, from almost one-fourth in 2010 to approximately 6% in 2020 (Figure 1B).

Fig 1. The number of available PubMed Central open access articles with “data/results not shown” phrase from dental (A) and all PubMed-indexed PMCOA (B) journals by publication year (bars), and percentage of articles with at least one “data/results not shown” phrase within each year (line).

In 2021, the search identified 1 951 unique PMCOA articles from 71 dental journals and 22 publishers. Of these, the search identified 57 PMCOA articles (from 20 different journals and six publishers), with at least one of either of the “data/results not shown” phrases. In the full-text review, all 57 were confirmed as including the phrase(s). Thus, 2.9% of the full texts in 2021 referred to unpublished results with the phrase. Fisher’s exact test showed a p-value of 0.03 for the journal-related, and 0.04 for the publisher-related differences in the percentages of studies with the phrase(s), from the total number of available PMCOA articles, from each journal or publisher in 2021 (Table 2 and Table 3, S4 Text).

Table 2. The number of available open access articles from Europe PubMed Central and the number of articles with “data/results not shown” phrases in each journal.

Journal	Publisher	Total number of studies	Studies with “data/results not shown”	Proportion (%) of studies with “data/results not shown”
BMC Oral Health	Springer Nature	470	15	3.2
Clinical Oral Investigations	Springer Nature	178	5	2.8
Journal of Applied Oral Science	Faculdade de Odontologia de Bauru	42	5	11.9
Clinical and Experimental Dental Research	John Wiley & Sons	72	3	4.2
International Journal of Oral Science	Springer Nature	29	3	10.3

The Journal of the American Dental Association	American Dental Association	31	3	9.7
Journal of Clinical Periodontology	John Wiley & Sons	15	3	20
Journal of Dental Research	SAGE Publishing	35	3	8.6
Odontology	Springer Nature	17	3	17.6
International Journal of Implant Dentistry	Springer Nature	93	2	2.2
Journal of Periodontology	John Wiley & Sons	6	2	33.3
Oral Diseases	John Wiley & Sons	80	2	2.5
The Angle Orthodontist	Allen Press Inc	96	1	1
Clinical Oral Implants Research	John Wiley & Sons	17	1	5.9
Head and Face Medicine	Springer Nature	41	1	2.4
International Endodontic Journal	John Wiley & Sons	5	1	20
Journal of Oral Rehabilitation	John Wiley & Sons	23	1	4.3
Journal of Periodontal Research	John Wiley & Sons	6	1	16.7
JDR Clinical and Translational Research	SAGE Publishing	19	1	5.3
Special Care in Dentistry	John Wiley & Sons	6	1	16.7
Others (51 journals)		670	0	0
Total		1951	57	2.9

Table 3. The number of available open access articles from Europe PubMed Central and the number of articles with “data/results not shown” phrases in journals of each publisher.

Publisher	Total number of studies	Studies with “data/results not shown”	Proportion (%) of studies with “data/results not shown”
Springer Nature	863	29	3.4
John Wiley & Sons	357	15	4.2
Faculdade de Odontologia de Bauru	42	5	11.9
SAGE Publishing	74	4	5.4
American Dental Association	31	3	9.7
Allen Press Inc	109	1	0.9
Others (16 publishers)	475	0	0

Of those 57 studies with the phrases, the phrases related most often to negative (n=17, 30%), peripheral (n=14, 24%) or confirmatory (n=9, 16%) results. Few referred to the sensitivity

analysis results (n=4, 7%). However, in some cases, it was difficult to evaluate their meaning or what results the phrase referred to (n=13, 23%). Twelve studies used the phrase(s) multiple times (eight studies two times, three studies three times, one study four times).

Some authors seemed to use the phrase just to indicate that certain numbers were not represented in the tables or figures but while providing data or results in the same sentence with words; for example: "The ratio of patients showing a history of head and neck cancer (19/47 vs. 14/97, $P = 0.0007$, data not shown)..." [14].

However, in some articles, notable conclusions were made based on data not shown. For instance, in a study investigating the association of preventive dental care to healthcare outcomes, tooth extractions and endodontic treatments were given considerable attention in terms of methodological decisions, results and their interpretation, but for restorative treatments, it was just stated in the discussion that, "Furthermore, we examined the effect of receiving restorative dental care on health outcomes, but no associations were seen (data not shown)" [15]. Other examples of how the phrases were used in the studies are provided in Table 1.

Twenty-four of the studies (42%) included a data and method sharing statement. Interestingly, one study used the raw statement template without any changes: "The data that support the findings of this study are openly available in [repository name e.g., "figshare"] at http://doi.org/[doi], reference number [reference number]" [16]. Only one shared data, and no study shared code, while 22 studies (39%) included supplementary material. The search from the journal websites of 20 journals that published at least one study with the phrase(s) showed that 17 would publish online supplementary materials

attached to the research articles for free, without limits. From three journals, we were unable to detect the information from their websites (The Angle Orthodontist, International Endodontic Journal, and Journal of Applied Oral Science).

Discussion

In agreement with promising trends in open science practices in biomedical studies over the last decades [5], we found that the proportion of PMCOA articles in dental journals that used the "data/results not shown" phrases had decreased significantly, from over 10% to approximately 3%, during the last decade. In 2021, of all PMCOA articles published until 29 September, 57 (2.9%) studies from 20 different journals used the "data/results not shown" phrases to refer to unpublished results. We found that there were differences in the use of the phrases between dental journals and publishers, where, most commonly, the phrases referred to negative, peripheral or confirmatory results. The significance of unpublished results to which the phrases referred varied considerably across studies. From those 57 studies, only one study shared data, and no study shared code.

Our findings showed a decreasing trend of PubMed-indexed PMCOA articles with the phrase "data/results not shown" from 1999 onwards. This trend, however, showed a steeper downtrend from 2008. This event occurred after the publication of data availability editorials in Nature journals, starting from 2006 [17–19]. Thereafter, several pieces of evidence tried to elaborate and express the concerns about data availability and reproducibility of results [20–23]. In 2016, these concerns were translated into a policy of a

mandatory statement on including information on whether and how others can access the underlying data for all research papers accepted for publication in Nature [24].

Many of the studied PMCOA articles were from open access dental journals, indexed in PubMed (BMC Oral Health and Clinical and Experimental Dental Research). Nieminen and Uribe [25] showed that in non-predatory (legitimate and indexed by established databases) open access dental journals, the presentation of results (particularly in tables and figures) was poorer than in more visible subscription-based dental journals, but still better than in predatory (non-indexed, from predatory publishers) dental journals. Since referring to “data not shown” evidently is an obscure way of presenting results, it may be related to how results are presented in these studies. Thus, poorer presentation of results in legitimate, open access journals may be one reason why such a high rate of the use of the phrases was detected in this study.

As we investigated only studies with freely available full texts, the high use of these phrases is a notable finding because it undermines some of the main arguments for open science [4–6]. Particularly, if researchers report a considerable number of results or important results with such phrases, and without sharing data or code, free interpretation and verification of results is doubly harder or even impossible, despite them being at the core of open science [4,5]. It has also been argued that the use of statistical significance thresholds threatens the reproducibility of scientific results [26]. It is evident that reporting negative results with “data/results not shown” phrases, which was still quite common in our sample in 2021, does not improve reproducibility.

Our results showed that the proportion of all PubMed-indexed PMCOA articles with “data/results not shown” phrases was considerably higher than in PubMed-indexed dental journals. Whereas the decreasing trend was evident in both, all PubMed-indexed PMCOA articles had a two-fold proportion in 2021 compared with dental journals. This potentially implies subject-related differences in the use of the phrases. So, investigating these differences in a further study could provide a better picture of the current situation.

Implications for research policy

Solutions to enhance the movement towards open science through abandoning “data/results not shown” can be postulated. The strictest solution could be banning the use of these phrases, accompanied by editorial requests for providing the data or results not shown. However, a more sustainable solution could be wider adoption of open science practices, as particularly free data and code sharing have remained rare in biomedical literature over the last decades [5]. In open access journals advocating for more open science [27,28], the obscure representation of results as well as not sharing data or code should not be overlooked by the publishers or editorial teams. The provided example in the Results section of this study shows that some editors may not even check the data availability statement in the papers. Thus, we need more commitment to open science principles from the editors.

Limitations

First, it is unknown how well PMCOA articles from dental journals represent the wider dental literature because subscription-based journals are underrepresented in the database of

open access articles. However, at least in terms of transparency indicators, the differences between PubMed-indexed and PMCOA articles might be small [29]. In addition, it is worth noting that the composition of the PMCOA database varies over time due to changes in open access practices and differences in how soon after publication, the journal's articles are made available to the database (ncbi.nlm.nih.gov/pmc/journals). Further, the total number of articles included all types of papers (commentaries, letters, etc.); hence, the proportion of studies with the phrase may be higher than what was found if solely research articles had been considered. Although the analysis of PMCOA articles published in 2021 showed that there were no false-positives in the search results (57 studies), we cannot be sure about the actual false-positive rates before 2021 or about the false-negative rate of our phrase identification strategy. Additionally, due to a large and heterogeneous sample of studies, we were unable to detect how the use of the phrases was related to other critical characteristics of studies, e.g., risks of bias.

Conclusion

We showed that a great decrease in the use of the selected phrases occurred in PMCOA articles published in PubMed-indexed dental and other journals over the last decades. However, dental or other researchers have not completely abandoned the outdated caveat of "data/results not shown", and it was still seen to be in use in 2021. Researchers, reviewers, editorial teams and publishers are responsible for further promoting and adopting open

science practices, including providing all results, data and code, whenever possible, in a freely accessible online format, one way or another; fortunately, it is possible today.

References

1. Goodman SN, Fanelli D, Ioannidis JPA. What does research reproducibility mean? Sci Transl Med. 2016 Jun 1;8(341):341ps12-341ps12.

2. McNutt M. Reproducibility. Science. 2014 Jan 17;343(6168):229–229.
3. Nosek BA, Alter G, Banks GC, Borsboom D, Bowman SD, Breckler SJ, et al. Promoting an open research culture. Science. 2015 Jun 26;348(6242):1422–5.
4. Munafò MR, Nosek BA, Bishop DVM, Button KS, Chambers CD, Percie du Sert N, et al. A manifesto for reproducible science. Nat Hum Behav. 2017 Jan;1(1):0021.
5. Serghiou S, Contopoulos-Ioannidis DG, Boyack KW, Riedel N, Wallach JD, Ioannidis JPA. Assessment of transparency indicators across the biomedical literature: How open is open? Bero L, editor. PLOS Biol. 2021 Mar 1;19(3):e3001107.
6. Vicente-Saez R, Gustafsson R, Van den Brande L. The dawn of an open exploration era: Emergent principles and practices of open science and innovation of university research teams in a digital world. Technol Forecast Soc Change. 2020 Jul;156:120037.
7. Camerer CF, Dreber A, Holzmeister F, Ho T-H, Huber J, Johannesson M, et al. Evaluating the replicability of social science experiments in Nature and Science between 2010 and 2015. Nat Hum Behav. 2018 Sep;2(9):637–44.
8. Leek JT, Jager LR. Is Most Published Research Really False? Annu Rev Stat Its Appl. 2017 Mar 7;4(1):109–22.
9. Nosek BA, Errington TM. Making sense of replications. eLife. 2017 Jan 19;6:e23383.
10. Naudet F, Sakarovitch C, Janiaud P, Cristea I, Fanelli D, Moher D, et al. Data sharing and reanalysis of randomized controlled trials in leading biomedical journals with a full data sharing policy: survey of studies published in *The BMJ* and *PLOS Medicine*. BMJ. 2018 Feb 13;k400.
11. Panter M. "Data Not Shown" - 4 Reasons to Omit a Figure or Table [Internet]. AJE Scholar. [cited 2021 Oct 13]. Available from: https://www.aje.com/arc/data-not-shown-4-reasons-omit-figure-or-table/
12. Data shown. Nat Chem Biol. 2008 Oct;4(10):575–575.
13. Wasserstein RL, Schirm AL, Lazar NA. Moving to a World Beyond " $p < 0.05$." Am Stat. 2019 Mar 29;73(sup1):1–19.
14. Yang S-W, Lee Y-S, Chang L-C, Yang C-H, Luo C-M, Wu P-W. Oral tongue leukoplakia: analysis of clinicopathological characteristics, treatment outcomes, and factors related to recurrence and malignant transformation. Clin Oral Investig. 2021 Jun;25(6):4045–58.
15. Lamster IB, Malloy KP, DiMura PM, Cheng B, Wagner VL, Matson J, et al. Dental

Services and Health Outcomes in the New York State Medicaid Program. J Dent Res. 2021 Aug;100(9):928–34.
16. Sereti M, Roy M, Zekeridou A, Gastaldi G, Giannopoulou C. Gingival crevicular fluid biomarkers in type 1 diabetes mellitus: A case–control study. Clin Exp Dent Res. 2021 Apr;7(2):170–8.
17. Nothing to hide. Nat Cell Biol. 2006 Jun;8(6):541–541.
18. Compete, collaborate, compel. Nat Genet. 2007 Aug;39(8):931–931.
19. Got data? Nat Neurosci. 2007 Aug;10(8):931–931.
20. Data producers deserve citation credit. Nat Genet. 2009 Oct;41(10):1045–1045.
21. It's not about the data. Nat Genet. 2012 Feb;44(2):111–111.
22. It's good to share. Nat Phys. 2014 Jul;10(7):463–463.
23. Laine C, Goodman SN, Griswold ME, Sox HC. Reproducible Research: Moving toward Research the Public Can Really Trust. Ann Intern Med. 2007 Mar 20;146(6):450.
24. Announcement: Where are the data? Nature. 2016 Sep;537(7619):138–138.
25. Nieminen P, Uribe SE. The Quality of Statistical Reporting and Data Presentation in Predatory Dental Journals Was Lower Than in Non-Predatory Journals. Entropy. 2021 Apr 16;23(4):468.
26. Amrhein V, Korner-Nievergelt F, Roth T. The earth is flat ($p > 0.05$): significance thresholds and the crisis of unreplicable research. PeerJ. 2017 Jul 7;5:e3544.
27. Tennant JP, Waldner F, Jacques DC, Masuzzo P, Collister LB, Hartgerink ChrisHJ. The academic, economic and societal impacts of Open Access: an evidence-based review. F1000Research. 2016 Sep 21;5:632.
28. Laakso M, Björk B-C. Anatomy of open access publishing: a study of longitudinal development and internal structure. BMC Med. 2012 Dec;10(1):124.
29. Wallach JD, Boyack KW, Ioannidis JPA. Reproducible research practices, transparency, and open access data in the biomedical literature, 2015–2017. Dirnagl U, editor. PLOS Biol. 2018 Nov 20;16(11):e2006930.
30. Hugo J, Koldsland OC, Aass AM, Tiainen H. Development and initial testing of an in vitro model simulating class II furcation defects. Clin Exp Dent Res. 2021 Apr;7(2):179–88.
31. Pagano S, Lombardo G, Costanzi E, Balloni S, Bruscoli S, Flamini S, et al. Morpho-functional effects of different universal dental adhesives on human gingival fibroblasts: an in vitro study. Odontology. 2021 Apr;109(2):524–39.
32. Sharma P, Fenton A, Dias IHK, Heaton B, Brown CLR, Sidhu A, et al. Oxidative stress links periodontal inflammation and renal function. J Clin Periodontol. 2021 Mar;48(3):357–67.
33. Kassem El Hajj H, Fares Y, Abou-Abbas L. Assessment of dental anxiety and dental phobia among adults in Lebanon. BMC Oral Health. 2021 Dec;21(1):48.
34. Örtengren U, Lehrkinder A, Safarloo A, Axelsson J, Lingström P. Opportunities for caries prevention using an ion-releasing coating material: a randomised clinical study. Odontology. 2021 Apr;109(2):358–67.

Supporting information captions

S1 Text. Deviations from the protocol

S2 Text. The searches for all available articles from dental journals in the Europe PubMed Central database on September 29, 2021.

S3 Text. The searches for articles with “data/results not shown” phrases in dental journals available from Europe PubMed Central database on September 29, 2021.

S4 Text. Sensitivity analysis for publisher-related differences

Fig 1

Click here to access/download;Figure;Fig 1.tif

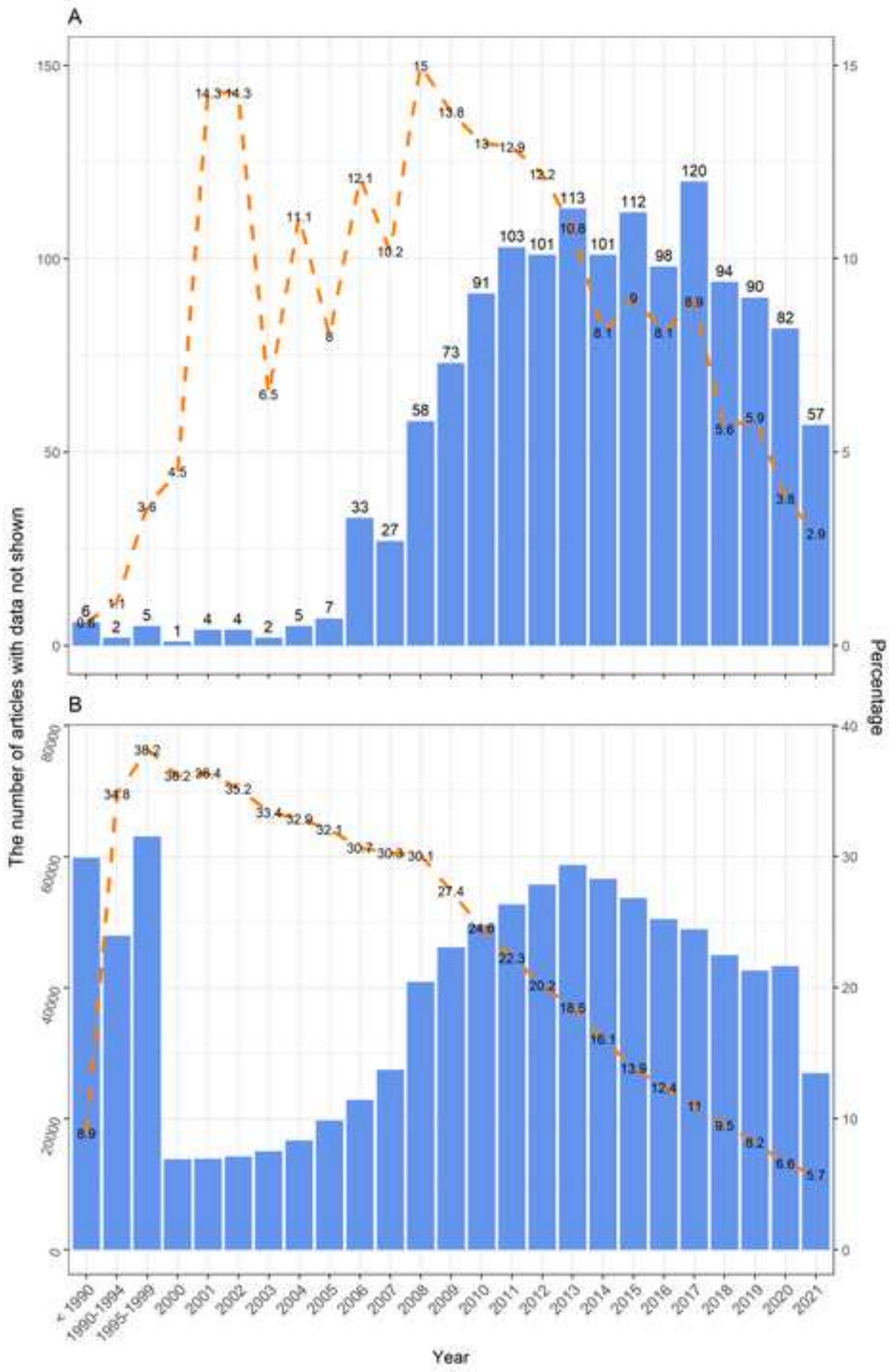

S1 Text

Click here to access/download
Supporting Information
S1 Text.docx

S2 Text

Click here to access/download
Supporting Information
S2 Text.docx

S3 Text

Click here to access/download
Supporting Information
S3 Text.docx

Click here to access/download
Supporting Information
S4 Text.docx
