# Supplementary material for: The use of the phrase “data not shown” in dental research
Source: PLoS One. 2022 Aug 9;17(8):e0272695. doi: 10.1371/journal.pone.0272695 (PMC9362922; doi:10.1371/journal.pone.0272695)
Supplement: Attachment — Submitted filename: Response to reviewers - PLOS ONE.docx [file pone.0272695.s004.docx]

Authors’ response: We thank the reviewers for their constructive and insightful comments. We see that the main reason for the rejection was that the reviewers didn’t consider the study worth conducting or publishing. We definitely agree with the reviewers that the importance of the study wasn’t well communicated in the submitted version.

First, what our study shows is what kinds of results the phrase “data not shown” refer to in the studies these days. We are unaware of any systematically conducted study that investigated what kind of unpublished results the phrases actually refer to. We see the investigation we made important for four reasons:

1) If researchers report a considerable amount of results or important results with such phrases, and without sharing results, data or code, free interpretation and verification of results are doubly harder or even impossible. In other words, the use of phrases obscures the transparency, reproducibility and weakens the peer review process.

2) As we showed, these phrases referred many times to non-statistically significant (negative) results. Focusing on statistically significant results and neglecting the negative ones is a major reason behind publication bias, but may also threaten the reproducibility of scientific results as argued by Amrhein et al (2017).

Amrhein V, Korner-Nievergelt F, Roth T. The earth is flat ( p > 0.05): significance thresholds and the crisis of unreplicable research. PeerJ. 2017 Jul 7;5:e3544.

3) Proper interpretation of sensitivity analyses require that modelling modifications, parameters and sensitivity results are adequately reported. As we showed, few papers referred to these kinds of results with the phrases.

Lash TL, Fox MP, MacLehose RF, Maldonado G, McCandless LC, Greenland S. Good practices for quantitative bias analysis. Int J Epidemiol. 2014 Dec;43(6):1969-85.

4) Results not considered important for the purposes of the current study may be crucial to conduct a systematic review or meta-analyses on the closely related topic. As we showed, many papers referred to peripheral (not directly related to the topic of study) or negative results with the phrases.

We have revised the introduction and discussion accordingly. We also made most of the revisions the reviewers suggested. We also made our analyses to cover the whole 2021 instead of including only part of 2021.

**Comments to the Author**

1. Is the manuscript technically sound, and do the data support the conclusions?

The manuscript must describe a technically sound piece of scientific research with data that supports the conclusions. Experiments must have been conducted rigorously, with appropriate controls, replication, and sample sizes. The conclusions must be drawn appropriately based on the data presented.

Reviewer #1: Yes

Reviewer #2: Yes

2. Has the statistical analysis been performed appropriately and rigorously?

Reviewer #1: Yes

Reviewer #2: I Don't Know

3. Have the authors made all data underlying the findings in their manuscript fully available?

The [PLOS Data policy](https://eur03.safelinks.protection.outlook.com/?url=http%3A%2F%2Fwww.plosone.org%2Fstatic%2Fpolicies.action%23sharing&data=04%7C01%7C%7C4c62bc79f51b4e316f6f08d9df7a4d4c%7C87879f2e73044bf2baf263e7f83f3c34%7C0%7C0%7C637786538794494791%7CUnknown%7CTWFpbGZsb3d8eyJWIjoiMC4wLjAwMDAiLCJQIjoiV2luMzIiLCJBTiI6Ik1haWwiLCJXVCI6Mn0%3D%7C3000&sdata=aIRm4%2Bu4nINkjo9Cn4o2XFcb4%2FbvkPsy%2FkvFmby5XNU%3D&reserved=0) requires authors to make all data underlying the findings described in their manuscript fully available without restriction, with rare exception (please refer to the Data Availability Statement in the manuscript PDF file). The data should be provided as part of the manuscript or its supporting information, or deposited to a public repository. For example, in addition to summary statistics, the data points behind means, medians and variance measures should be available. If there are restrictions on publicly sharing data—e.g. participant privacy or use of data from a third party—those must be specified.

Reviewer #1: Yes

Reviewer #2: No

Authors’ response: All data and code produced and analyzed during the study are shared via Open Science Framework <https://osf.io/5zryu/>

4. Is the manuscript presented in an intelligible fashion and written in standard English?

PLOS ONE does not copyedit accepted manuscripts, so the language in submitted articles must be clear, correct, and unambiguous. Any typographical or grammatical errors should be corrected at revision, so please note any specific errors here.

Reviewer #1: Yes

Reviewer #2: Yes

5. Review Comments to the Author

Please use the space provided to explain your answers to the questions above. You may also include additional comments for the author, including concerns about dual publication, research ethics, or publication ethics. (Please upload your review as an attachment if it exceeds 20,000 characters)

Reviewer #1: The reviewer's concerns were expressed in the attached marked-up PDF document. The authors are required to better justify the necessity of this research, given the actual possibilities of a reviewer to require all available data/results, when needed.

[We extracted the comments from the PDF:]

Although originating in the text of an authorless note (editorial ?) in Nat Chem Biol, the title seems pretentious and bombastic. This is a study of scientometrics, not a chapter of a mystery novel. Besides, the meaning is not clear. As far as the reviewer knows, a "caveat" means "warning", "caution". As such, a caveat cannot be characterized neither as "fashionable", nor as "outdated". Even more, the results of the research show clearly that the mention "data not shown" seems not to be outdated, but rather reduced as frequency of use. Please try to modify the title as to reflect the content of the research.

Authors’ response: We edited the title.

The phrase is unclear. Please reformulate.

Authors’ response: The sentence was reformulated.

This source-phrase sounds pretentious, and, at a closer look, is trivial. Hidden data always affect the transparency of a research and weakens the peer review.

Authors’ response: We reformulated the sentence.

Please justify the necessity of such a research. At what extent so far has actually the phrase "data/results not shown" produced damages in terms of interpreting the results and drawing sound conclusions? It si still not clear why this research was needed.

Authors’ response: We agree with the reviewer that the importance of the study wasn’t elaborated clearly enough. Please see the beginning of the response,

OSF means...?

Authors’ response: Open Science Framework, it was stated in the introduction.

Please explain why 2021 was chosen as a year of reference?

Authors’ response: Most recent year. Current use of the phrase is most interesting because every day sharing data and other material becomes easier and easier.

How does apply this statement to the present research?

Authors’ response: The sentence was removed.

In the Discussion section, authors should investigate the significance of the mention "data not shown" in the context of an undeterrable request for data from the reviewers. In other words, how effective could be the mention "data/results not shown", knowing that these data/results can be always requested by the reviewers?

Authors’ response: This is now mentioned in the limitations section.

Based on the distribution of the hidden data/results, in the Discussion section, authors should further speculate what could be the real reason(s) for not showing the data/results.

Authors’ response: We added a paragraph about the reasons for the use of the phrase.

Since discussing the reasons: the reviewer's experience shows that in many cases less relevant data are poorly presented/not shown, and this is not even stated as such. Given this reality, could the mention "data/results not shown" be seen as a sign of ultimate, twisted honesty from the part of the authors?

Authors’ response: This is now stated in the paragraph we added to the discussion.

It is not clear what "false-positive" or "false-negative" means in this context. Please explain.

Authors’ response: This was clarified.

Reviewer #2: Dear authors,
The Manuscript ID PONE-D-21-34697 entitled " Movement towards open science: Have dental researchers abandoned the outdated caveat “data not shown”?" analyses the evolution in time of the use of the phrases “data/results not shown” and to what kind of results the authors referred to with these phrases in 2021.
Although the paper is beautifully written and has few changes to be made, I am afraid I don´t find the interest in the topic. The conclusions reached add little innovation and, although I think editors should really handle this information, I don´t know if readers will get much from it.

Authors’ response: We agree with the reviewer that the importance of the study wasn’t elaborated clearly enough. Please see the beginning of the response,

Specifically, there are some mistakes in the text that should be corrected:
- What do authors mean by “open access”? Some of the indexed journals included in the sample are not available in open access, only subscription-based; I am afraid I don´t really understand how you made the selection of journals and editors.

Authors’ response: All PubMed-indexed dental journals were selected based on a list provided by National Library of Medicine (NLM). Open access in this context means that the article is freely accessible via Europe PubMed Central database. Therefore, the study sample also includes studies from subscription-based journals.

- Page 4 line 44: There is a missing point after the (1-4)
- Page 6 line 98: the citation is badly spelled “m [11)”.
- The authors establish a comparison between their results from 2021 and previous data. Nevertheless, I don´t get to find in the text the original source from where you achieved previous figures. Allusions to the trends in the past are made and cited in the Discussion, but not in the Results, where the authors first make the comparison.

Authors’ response: These were corrected to manuscript.

- Page 11 line 160: the use of the adverb “Interestingly” reads sarcastic and complicates the understanding of the statement.

Authors’ response: This was removed.
- The quality of the figures is quite poor; I don´t know if it would be possible to upload them in higher resolution.

Authors’ response: Figures were improved.
