# Supplementary material for: The use of the phrase “data not shown” in dental research
Source: PLoS One. 2022 Aug 9;17(8):e0272695. doi: 10.1371/journal.pone.0272695 (PMC9362922; doi:10.1371/journal.pone.0272695)
Supplement: Attachment — Submitted filename: Response to reviewers_rev2.docx [file pone.0272695.s005.docx]

We thank the editor and reviewers for the opportunity to submit our revised manuscript.

The comments of reviewer 3 should be considered to improve the article.

My main concern is about including only studies published in 2021. Authors should highlight in the discussion why this decision and what is the impact of that.

Response: Our aim was to investigate reasons for current use of the phrases, because nowadays researchers have many options to share data and results freely and easily. We didn’t see it worthwhile investigating the use of phrases in more detail from the whole sample, because to some extent the use of phrases has been acceptable (for instance before widespread use of the internet).

Reviewer #3: This is a well-written paper that addressed how frequently papers published in dental journals use the phrases “data/results not shown" and what kind of results the authors referred to with these phrases in 2021. Some study limitations cannot be disregarded, especially the underrepresentation of subscription-based journals and the lack of information on the true reasons for using such phrases. Despite that, I acknowledge that the exploratory nature of these findings can give us insightful information regarding open access practices in dental journals.

Response: We thank the reviewer for his/her insightful comments.

The authors have substantially improved the first version of the paper. However, I strongly suggest authors change the title. Surely the authors made a systematic search. However, this was not a systematic review, but a scientometric analysis.

Response: We thank the reviewer for this good comment. We removed the type of study from the title completely: The use of the phrase “data not shown” in dental research

Also, the authors should explain why the protocol for this study was not prospectively registered.

Response: The reviewer is right that the protocol was not registered beforehand, it was just shared publicly beforehand. That was our fault, we deemed it acceptable to just publish it beforehand instead of using registers such as those available via Open Science Framework. However, this doesn’t change the fact that we have worked closely following the open science principles when conducting this study.

Another comments:

Introduction, last paragraph: “Accordingly, from relevant studies in 2021, we examined….” Please consider replacing the word relevant with eligible.

Response: We edited the sentence in this regard, but also to highlight why we conducted in more detail analysis of those articles that used the phrases in 2021.

Material and methods: the topic “study selection” should be rewritten and divided into three topics: “protocol registration”, “bibliographic search”, and “eligibility criteria” (or similar topics). Information regarding these three topics is all mixed in “study selection”, and the reader may find it difficult to understand.

Response: We thank the reviewer for pointing this out. We edited and added more subsections with more detail subheaders: “protocol registration”, “bibliographic search”, “Selection of 2021 subsample”, “Data extraction from the 2021 subsample” and “Data synthesis and analyses“)

Results: “The percentage of studies with the phrases from all the PMCOA articles decreased from 13% to approximately 3% in dental journals from 2010 to 2020 (Figure 1A)”. It is not clear why the years 2010 and 2020 were chosen.

Response: We thank the reviewer for pointing this out. We improved the whole paragraph and added a more detailed description of our findings:

“As the total number of PMCOA dental articles was low before 2010, there was considerable fluctuation in the percentage of studies with the phrases. As the number of available dental articles increased, the percentage of studies with the phrases stabilised to around 13% by 2010. From 2010, the percentage decreased to approximately 3% in dental journals from 2010 to 2020 (Figure 1A).

Amongst all PubMed-indexed PMCOA articles the percentages were clearly higher in all study years than in dental journals. The percentage of articles with the phrases decreased from 1990 (35%) to 2020 (6%) (Figure 1B). In both samples, these trends showed a steeper downtrend from the late 2000s onwards.”

In S4 Text (Sensitivity analysis for publisher-related differences), please provide the raw numbers and not only the p values.

Response: We thank the reviewer for pointing this out. Raw numbers for journal- and publisher-related differences are now provided in supplementary material available at https://osf.io/5zryu/.
